# Balance Problems, Paralysis, and Angina as Clinical Markers for Severity in Major Depression

**DOI:** 10.3389/fpsyt.2020.567394

**Published:** 2020-12-23

**Authors:** Bill Qi, Kellie MacDonald, Marcelo T. Berlim, Allan Fielding, Eric Lis, Nancy Low, Stéphane Richard-Devantoy, Valerie Tourjman, Gustavo Turecki, Yannis Trakadis

**Affiliations:** ^1^Department of Human Genetics, McGill University, Montreal, QC, Canada; ^2^Neuromodulation Research Clinic, Depressive Disorders Program, Douglas Mental Health University Institute, McGill University, Montreal, QC, Canada; ^3^Department of Psychiatry, McGill University, Montreal, QC, Canada; ^4^McGill University Psychiatry Perceptions of Emerging Technologies Labs, Montreal, QC, Canada; ^5^McGill Group for Suicide Studies, Douglas Mental Health University Institute, McGill University, Montreal, QC, Canada; ^6^Institut Universitaire en Santé Mentale de Montréal, Montreal, QC, Canada; ^7^Department of Medical Genetics, McGill University Health Center, Montreal, QC, Canada

**Keywords:** biomarkers, depression, severity, symptoms, stratification

## Abstract

Major depressive disorder (MDD) is a heterogeneous disorder. Our hypothesis is that neurological symptoms correlate with the severity of MDD symptoms. One hundred eighty-four outpatients with MDD completed a self-report questionnaire on past and present medical history. Patients were divided into three roughly equal depression severity levels based on scores from the APA Severity Measure for Depression—Adult (*n* = 66, 58, 60, for low, medium, high severity, respectively). We saw a significant and gradual increase in the frequency of “muscular paralysis” (1.5–5.2–16.7%) and “balance problems” (21.2–36.2–46.6%) from low to medium to high severity groups. We repeated the analysis using only the two most extreme severity categories: low severity (66 samples) vs. high severity (60 samples). High severity patients were also found to experience more “angina” symptoms than low severity patients (27.3 vs. 50%). The three significant clinical variables identified were introduced into a binary logistic regression model as the independent variables with high or low severity as the dependent variable. Both “muscular paralysis” and “balance problems” were significantly associated with increased severity of depression (odds ratio of 13.5 and 2.9, respectively), while “angina” was associated with an increase in severity with an odds ratio of 2.0, albeit not significantly. We show that neurological exam or clinical history could be useful biomarkers for depression severity. Our findings, if replicated, could lead to a simple clinical scale administered regularly for monitoring patients with MDD.

## Introduction

Major depressive disorder (MDD) is characterized by one or more major depressive episodes (MDEs) and the absence of mania and hypomania throughout an individual's lifetime (1). An MDE includes depressed mood, loss of interest or pleasure, change in weight or appetite, sleep disturbances, psychomotor problems, fatigue, worthlessness or guilt, impaired concentration or indecisiveness, and thoughts of death or suicide (2). Negative outcomes in depression, such as suicidal behavior, highlight the importance of early diagnosis and treatment (3). For an individual to be diagnosed with MDD, at least five symptoms need to be present within a period of 2 weeks. Of these five symptoms, depressed mood or loss of interest and pleasure must be present for a diagnosis of MDE to be made.

MDD is a complex and heterogeneous disorder with a wide range of risk factors, severity, and treatment response. Several studies have shown that behavioral and cognitive phenotypes can be useful for biomarker discovery in MDD. Taylor et al. (4) reported that psychomotor slowing was predictive of poor response to fluoxetine, while Gorlyn et al. (5) showed how global cognitive functioning can serve as a marker for predicting selective serotonin reuptake inhibitors (SSRI) treatment response. Lastly, a recent study has shown that movement data collected from wearable devices had a high correlation with depression severity (6).

Depression is encountered in different neurological disorders and idiopathic MDD and “neurologic” depression seem to share common abnormalities in specific brain areas (7). For instance, depressive symptoms have been well-documented in patients with stroke (8), epilepsy (9), multiple sclerosis (10), and dementia (11). According to Gutzmann et al. (2015), the severity of depression increases with increasing severity of neurological impairments. Similarly, Smith et al. (12) found that cognitive performance in individuals with prodromal Huntington disease is related to depressive symptom severity. Moreover, very mild depressive symptoms have also been shown to be associated with gait disturbance in early Parkinson's disease (PD) and it has been hypothesized that depression may influence mechanisms of gait disturbance (13). This hypothesis is in line with the results of a multicenter randomized study showing that gait instability (freezing of gait), in patients with PD, responds to treatment with antidepressants (14). Our hypothesis is that neurological symptoms correlate with the severity of the depression symptoms. This study explores whether any findings in non-psychiatric past medical history correlate with depression severity, potentially allowing their use as biomarkers for the prognosis and monitoring of patients with depression.

## Methods

### Participant Recruitment and Data Collection

The study protocol was approved by the Research Ethics Board of the Douglas Mental Health University Institute (DMHUI), the McGill University Health Centre (MUHC) and the Institut Universitaire en Santé Mentale de Montréal (IUSMM). One hundred eighty-four consecutive, unselected patients with major depression, between the ages of 19 and 77 years, were recruited from tertiary outpatient depression clinics at the DMHUI, Allan Memorial Institute (AMI), and IUSMM. All patients were diagnosed by certified psychiatrists, using the the Diagnostic and Statistical Manual of Mental Disorders, 5th edition (DSM-5). A self-report questionnaire screening for non-psychiatric medical symptoms was given to all study participants after providing written informed consent. This questionnaire consisted of 49 individual questions relating to distinct categorical clinical variables. It was used to survey participants on their past and present medical history, as well as that of their family and their educational history. We also surveyed participants for psychiatric medication use.

Finally, a self-report questionnaire called the APA Severity Measure for Depression—Adult [adapted from the Patient Health Questionnaire−9 (PHQ-9)] (15, 16) was used to evaluate the severity of each participant's condition. The APA Severity Measure for Depression that was used was developed by the American Psychiatric Association. It was adapted from the Patient Health Questionnaire−9 (PHQ-9), which has been shown to be a reliable and valid test for documenting the severity of depression. This measure was chosen, as it provides more detailed instructions for scoring and interpretation than the PHQ-9, while maintaining the same questions and general marking scheme as the PHQ-9.

### Statistical Method

Participants were divided into three severity categories based on their depression severity scores: (1) low severity, scores range from 1 to 12 (66 individuals), (2) medium severity, scores range from 13 to 18 (58 individuals), and 3) high severity, scores above 18 (60 individuals). Forty-nine categorical clinical variables, including age, sex, education level, neurological features, family history, dietary and gastrointestinal features, cardiovascular features, and other clinical features were analyzed with the chi-square test in contingency tables (χ2). The age variable was analyzed using a Student's *t*-test. Significance was set at *P* ≤ 0.05 (two-tailed). We then repeated the analyses using only the two most extreme severity categories: low severity (66 samples) vs. high severity (60 samples).

Ordinal logistic regression was applied to determine the contributions of the significant variables from the chi-square tests in discriminating low vs. medium vs. high severity depression patients. Variables meeting the significance cut-off from the chi-square test for the three severity categories analysis were used as input variables for the model. Similarly, binary logistic regression was applied to determine the contributions of the significant variables in discriminating low vs. high severity depression patients.

Lastly, a linear regression was applied to model the relationship between the number of psychiatric medications used by patients and their depression severity score. We excluded outlier patients who had more than 6 medications based on a boxplot of medication counts (Supplementary Figure 1).

We also calculated the percentage of patients receiving each specific medication in the high vs. low severity category and identified the medications with the largest differences among the two groups. We then examined the side-effects of these medications and verified whether they were related to any significant clinical variables identified.

The chi-square tests were performed using the *scipy* python package (ver. 1.1.0), while the binary logistic regression and linear regression analyses were performed using the *statsmodels* python package (ver. 0.10.1). The ordinal logistic regression analysis was performed using the *MASS* R package (ver. 7.3-51.3).

## Results

For the three severity categories analysis, a significant difference between observed and expected frequencies was found for the “muscular paralysis” and “balance problems” variables. Of note, as part of our questionnaire these two terms were described as “muscular paralysis (e.g., complete loss of muscle function)” and “problems with balance and/or movement coordination (e.g., falls, bumping into objects, short repeated episodes or progressive episodes).” Low, medium, and high severity categories had patients with “muscular paralysis” with frequencies 1.5, 5.2, and 16.7%, and “balance problems” with frequencies 21.2, 36.2, and 46.6%, respectively.

We then repeated the analysis with two severity categories. A significant difference was found again for “muscular paralysis,” “balance problems,” but also for “angina” (defined as “chest pain and/or chest tightness”). The low severity category had 27.3%, while the high severity category had 50% of patients with “angina.”

The significant clinical variables (“muscular paralysis” and “balance problems”) identified from the three-severity category analysis were introduced into an ordinal logistic regression model with severity (ordered from high, medium, to low) as the dependent variable. Both “muscular paralysis” and “balance problems” were significantly associated with increased severity of depression with an odds ratio of 6.5 (*p* = 0.0022) and 2.5 (*p* = 0.0024) respectively (Table 1).

**Table 1:** Results from the ordinal logistic regression for the significant variables identified from the chi-square tests with patients from the high, medium, or low depression severity categories.

**Significant variables from chi-square tests**	**Coefficient**	**Standard Error**	***t***	***p***	**OR**	**OR 2.5%**	**OR 97.5%**
Muscular paralysis	1.878	0.613	3.064	0.0022	6.541	2.115	24.767
Balance problems	0.900	0.296	3.037	0.0024	2.458	1.383	4.424

*An ordinal logistic regression contrasting the 60 high, 58 medium, and 66 low depression severity patients was performed with the significant variables from the chi-square tests (“Muscular paralysis,” and “Balance problems”). The table details the model coefficients, standard errors, t-values, and p-values of the coefficients, as well as the odds ratio (OR) and the 95% confidence interval (CI) (OR 2.5%, OR 97.5%) derived from the coefficient and standard errors. The coefficient for both “Muscular paralysis” and “Balance problems” were significantly different from zero (p < 0.05)*.

Similarly, the significant clinical variables (“muscular paralysis,” “balance problems,” and “angina”) identified from the two severity categories were introduced into a binary logistic regression model as the independent variables with high or low severity as the dependent variable. After obtaining the optimal fit, the model accounted for 13.0% of the variance (*p* = 4.8^*^10^−5^). Both “muscular paralysis” and “balance problems” were significantly associated with increased severity of depression with an odds ratio of 13.5 (*p* = 0.016) and 2.9 (*p* = 0.011), respectively, while “angina” was associated with an increase in severity with an odds ratio of 2.0, albeit not significantly (*p* = 0.092) (Table 2).

**Table 2:** Results from the binary logistic regression for the significant variables identified from the chi-square tests with patients from the high or low depression severity categories.

**Significant variables from chi-square tests**	**Coefficient**	**Standard Error**	**Z**	***p***	**OR**	**OR 2.5%**	**OR 97.5%**
Muscular paralysis	2.606	1.086	2.400	0.016	13.546	1.613	113.787
Balance problems	1.074	0.424	2.534	0.011	2.926	1.275	6.714
Angina	0.692	0.411	1.683	0.092	1.997	0.892	4.469
Intercept	−0.885	0.274	−3.224	0.001	0.413	0.241	0.707

*A binary logistic regression contrasting the 60 high and 66 low depression severity patients was performed with the significant variables from the chi-square tests (“Muscular paralysis,” “Balance problems,” and “Angina”). The table details the model coefficients, standard errors, z-values (Z), and p-values of the coefficients, as well as the odds ratio (OR) and the 95% confidence interval (CI) (OR 2.5%, OR 97.5%) derived from the coefficient and standard errors. The coefficient for both “Muscular paralysis” and “Balance problems” were significantly different from zero (p < 0.05), while the coefficient for “Angina” was borderline significant. The 95% CI is large for “Muscular paralysis,” suggesting that a larger sample size would be required to have a more precise estimate of its effect on depression severity. Of note, the intercept is showing the odds of being severely depressed if someone did not have any features of “muscular paralysis”, “balance problems” or “angina”*.

Lastly, we applied linear regression with the number of psychiatric medications of each patient as the independent variable and the depression score as the dependent variable. A table of the list of medications we considered to be psychiatric is available in Supplementary Table 1. One patient was removed as an outlier based on the boxplot of the number of medications (Supplementary Figure 1). After obtaining the optimal fit, the model only explained 0.4% of the variance (*p* = 0.40) (Supplementary Figure 2) of the depression score. For every medication, the percentage of patients receiving it in the high vs. low severity category is shown in Supplementary Table 1. Bupropion and sertraline, were taken by a larger proportion of high severity category patients (18 and 12%) compared to low severity category patients (11 and 2%), however, no medication side-effects were found to be related to “balance problems,” “muscular paralysis,” or “angina” for either medication.

## Discussion

This study explores whether any findings in non-psychiatric past medical history correlate with depression severity. Our hypothesis was that neurological symptoms correlate with the severity of the depression symptoms, potentially allowing their use as clinical biomarkers for the prognosis and monitoring of patients with depression. Our results show that more severely depressed patients have a higher likelihood for neurological and cardiovascular symptoms. More specifically, “muscular paralysis” and “balance problems” are associated with increasing depression severity (*p* < 0.05). Similarly, some evidence was found for “angina,” albeit not meeting our cut-off for significance (*p* = 0.092).

One possible explanation is that as the severity of depression increases, so does the number of medications, leading to side-effects reported as symptoms. However, this does not seem to be the case in our study based on the regression analysis performed for the number of medications vs. severity, and based on the side-effects profile of the medications that were taken by a higher percentage of high severity patients compared to low severity patients. In brief, we found no significant differences between age, sex, education levels, or the number of psychiatric medications taken between patients with different severity of depression.

To explore the possibility that pathophysiological changes underly both depressive and neurological symptoms, a literature review was performed to search for existing evidence supporting a link. We found evidence for “balance problems” or ataxia in MDD. For example, a slow walk with reduced arm swinging and a more slumped posture are characteristic of depression (17). Moreover, an association of MDD with falls has been published by different studies (18). Studies have shown a significantly smaller vermis in patients with MDD without ataxia (19), and smaller cerebellum in patients with bipolar disorder (20). These brain structures are known to be important in equilibrium and coordination. Of note, patients with cerebellar dysfunction show higher scores on depression inventories when compared to controls (21). Interestingly, in some genetic conditions characterized by ataxia, depression appears to be an important feature. For example, a recent study found that 57% of patients with spinocerebellar ataxia type 3 (SCA3) had depression and that this seemed to have a significant impact, positively contributing to the severity of their ataxia (22). Similarly, in a study of patients with Friedreich's ataxia, 21% of participants were found to have depression in the moderate/severe range (23).

A similar search was performed for “muscular paralysis.” Szklo-Coxe et al. (24) found that having severe depression lead to a 500% increase in the odds of having sleep paralysis (24). It has also been shown that leaden paralysis may be common in atypical depression, with one study reporting 47% of their patients with atypical depression presenting with leaden paralysis (25). Of note, leaden paralysis is not referring to a real “muscular paralysis (e.g., complete loss of muscle function).” Rather, it consists of severe fatigue creating a sensation of extreme heaviness of the arms or legs and it is considered a reliable marker of atypical depression.

Finally, a link between “angina,” as well as other cardiac conditions, and depression, is well-established. MDD is a risk factor for cardiovascular disease (CVD), even after adjusting for demographics and traditional cardiovascular risk factors (26). In a longitudinal study of a cohort of patients without CVD at baseline, it was determined that depression was significantly associated with the incidence of a cardiac event and that this was unlikely to be due to the effects of hypertension, diabetes, or dyslipidemia. Of the 592 persons who experienced a cardiac event in this study, 160 were classified as “angina” (27). Additionally, an increase in PHQ-9 depression severity scores have been associated with an increase in “angina” frequency, thus validating our finding; further, newly depressed individuals have been shown to report more “angina” than those who do not have depression (28). Moreover, a study assessing “angina” in patients with MDD and coronary artery disease found that having depression predisposed an individual to a greater risk of “angina” and that the severity of their coronary artery disease did not seem to impact this (29). Finally, a recent study found that symptoms of chest tightness/chest pain were predictors of the onset of symptoms of depression and anxiety in patients that had been recently referred to neurology outpatient clinics (30), which further supports the findings of our current study. In conclusion, we provide evidence that non-psychiatric clinical symptoms, including neurological features, can serve as clinical markers for disease severity. Our findings, if replicated, could lead to a simple clinical scale administered regularly for monitoring patients with MDD based on review of systems and/or physical examination.

## Limitations and Future Directions

One of the limitations of this study is the relatively small sample size. Our findings should be replicated in the future with larger sample sizes, which are adequately powered to explore interactions between variables, and maybe capture other potentially relevant patient populations (e.g., hospitalized patients with MDD and/or patients with bipolar disorder). Moreover, in our study, the PHQ-9 self-report questionnaire was used. It would be important for future studies to consider adding objective assessments by trained personnel to ensure that the data collected is more standardized between study participants. Patients in our study were recruited from different tertiary depression clinics and, although all patients met DSM-5 criteria for MDD, there was no uniform use of a structural instrument as part of their evaluation. Future studies could consider using a structural instrument such as the Mini International Neuropsychiatric Interview (MINI) or the Structured Clinical Interview for DSM (SCID).

Most importantly, our questions for the significant features were asking the patients if they “experience short episodes of chest pain and/or chest tightness (also known as angina)?” or “problems with balance and/or movement coordination (e.g., falls, bumping into objects, short repeated episodes or progressive episodes)” or “muscular paralysis (e.g., complete loss of muscle function).” However, there was no clear question allowing temporal qualification, and this was one of the limitations of our study that future studies need to address. Close monitoring of temporal relationship of the clinical markers identified in our study to the depressive symptoms is important. It can validate the importance of these clinical markers for monitoring of MDD and potentially ensure adjustment in the antidepressant regimen.

A prospective study focused on targeted medical history for these features, along with physical examination for cerebellar findings, would be needed to explore if changes in these features precede the subjective experience of worsening symptomatology of MDD. If, indeed, our findings are replicated and subtle changes on the neurological examination or clinical history are proven to be useful clinical markers of changes in depression severity, our findings could lead to a simple clinical scale administered on a regular basis, along with the validated neuropsychiatric tools already in use, for monitoring patients with MDD. Ultimately, this could result in earlier intervention in patients with depression, enabling the physician to adjust the treatment regimen before the depressive symptoms become very severe. This could potentially help us optimize pharmacological interventions and reduce negative outcomes, such as suicide.

## Data Availability Statement

The raw data supporting the conclusions of this article will be made available by the authors, without undue reservation.

## Ethics Statement

The studies involving human participants were reviewed and approved by the Research Ethics Board of the Douglas Mental Health University Institute (DMHUI), the McGill University Health Centre (MUHC) and the Institut Universitaire en Santé Mentale de Montréal (IUSMM). The patients/participants provided their written informed consent to participate in this study.

## Author Contributions

BQ performed the statistical analyses and drafted the manuscript under the supervision of YT who conceived and coordinated the project. KM performed quality check and pre-processing of the data, and the literature reviews and coordinated patient recruitment, and data collection. BQ and YT designed the original methodology. MB, AF, EL, NL, SR-D, VT, and GT are MDs who actively engaged in patient recruitment. All authors reviewed and provided feedback on the manuscript.

## Conflict of Interest

The authors declare that the research was conducted in the absence of any commercial or financial relationships that could be construed as a potential conflict of interest.

## References

[1] Uher R Payne JL Pavlova B Perlis RH. Major depressive disorder in DSM-5: implications for clinical practice and research of changes from DSM-IDepression V. and Anxiety. (2014) 31:459–71. 10.1002/da.22217 24272961

[2] American Psychiatric Association Diagnostic and statistical manual of mental disorders. BMC Med. (2013) 17:133–7. 10.1176/appi.books.9780890425596

[3] Pompili M Innamorati M Lamis DA Erbuto D Venturini P Ricci F. The associations among childhood maltreatment, “male depression” and suicide risk in psychiatric patients. Psychiatr Res. (2014) 220:571–8. 10.1016/j.psychres.2014.07.056 25169890

[4] Taylor BP Bruder GE Stewart JW McGrath PJ Halperin J Ehrlichman H. Psychomotor slowing as a predictor of fluoxetine nonresponse in depressed outpatients. Am J Psychiatr. (2006) 163:73–8. 10.1176/appi.ajp.163.1.73 16390892

[5] Gorlyn M Keilp JG Grunebaum MF Taylor BP Oquendo MA Bruder GE. Neuropsychological characteristics as predictors of SSRI treatment response in depressed subjects. J Neural Trans. (2008) 115:1213. 10.1007/s00702-008-0084-x 18629432 PMC3767987

[6] Jacobson NC Weingarden H Wilhelm S. Using digital phenotyping to accurately detect depression severity. J Nervous Mental Dis. (2019) 207:893–6. 10.1097/NMD.0000000000001042 31596769

[7] Benedetti F Bernasconi A Pontiggia A Depression and neurological disorders. Curr Opin Psychiatr. (2006) 19:14–8. 10.1097/01.yco.0000194147.88647.7f 16612173

[8] Pfeil M Gray R Lindsay B. Depression and stroke: a common but often unrecognized combination. Br J Nurs. (2009) 18:365–9. 10.12968/bjon.2009.18.6.40769 19329901

[9] Carrieri PB Provitera V Iacovitti B Iachetta C Nappi C Indaco A. Mood disorders in epilepsy. Acta Neurol. (1993) 15:62–7. 8456597

[10] Ehde DM Bombardier CH Depression in persons with multiple sclerosis. Phys Med Rehabil Clinics North Am. (2005) 16:437–48. 10.1016/j.pmr.2005.01.010 15893680

[11] Gutzmann H Qazi A. Depression associated with dementia. Zeitschrift für Gerontol Geriatr. (2015) 48:305–11. 10.1007/s00391-015-0898-8 25962363

[12] Smith MM Mills JA Epping EA Westervelt HJ Paulsen JS. Depressive symptom severity is related to poorer cognitive performance in prodromal Huntington disease. Neuropsychology. (2012) 26:664–9. 10.1037/a0029218 22846033 PMC3806339

[13] Lord S Galna B Coleman S Burn D Rochester L. Mild depressive symptoms are associated with gait impairment in early Parkinson's disease. Movement Disord. (2013) 28:634–9. 10.1002/mds.25338 23390120

[14] Takahashi M Tabu H Ozaki A Hamano T Takeshima T. Antidepressants for depression, apathy, and gait instability in Parkinson's disease: a multicenter randomized study. Int Med. (2019) 58:361–8. 10.2169/internalmedicine.1359-18 30146591 PMC6395136

[15] Kroenke K Spitzer RL Williams JBW. The PHQ-9: validity of a brief depression severity measure. J General Int Med. (2001) 16:606–13. 10.1046/j.1525-1497.2001.016009606.x 11556941 PMC1495268

[16] Severity Measure for Depression—Adult (2019). Available online at: https://www.psychiatry.org/File%20Library/Psychiatrists/Practice/DSM/APA_DSM5_Severity-Measure-For-Depression-Adult.pdf.~

[17] Michalak J Troje NF Fischer J Vollmar P Heidenreich T Schulte D. Embodiment of sadness and depression—gait patterns associated with dysphoric mood. Psychosomat Med. (2009) 71:580–7. 10.1097/PSY.0b013e3181a2515c 19414617

[18] Deandrea S Lucenteforte E Bravi F Foschi R La Vecchia C Negri E Risk factors for falls in community-dwelling older people: “a systematic review and meta-analysis”. Epidemiology. (2010) 658–68. 10.1097/EDE.0b013e3181e89905 20585256

[19] Yucel K Nazarov A Taylor VH Macdonald K Hall GB MacQueen GM. Cerebellar vermis volume in major depressive disorder. Brain Struct Funct. (2013) 218:851–8. 10.1007/s00429-012-0433-2 22696069

[20] Brambilla P Barale F Caverzasi E Soares JC. Anatomical MRI findings in mood and anxiety disorders. Epidemiol Psychiatr Sci. (2002) 11:88–99. 10.1017/S1121189X00005558 12212470

[21] Clausi S Lupo M Olivito G Siciliano L Contento MP Aloise F. Depression disorder in patients with cerebellar damage: awareness of the mood state. J Affect Disord. (2019) 245:386–93. 10.1016/j.jad.2018.11.029 30423466

[22] Lin MT Yang JS Chen PPZ Lin HX Chen XP. Bidirectional connections between depression and ataxia severity in spinocerebellar ataxia type 3 patients. Eur Neurol. (2018) 79:266–71. 10.1159/000489398 29763923

[23] Nieto A Hernández-Torres A Pérez-Flores J Montón F. Depressive symptoms in Friedreich ataxia. Int J Clin Health Psychol. (2018) 18:18–26. 10.1016/j.ijchp.2017.11.004 30487906 PMC6220911

[24] Szklo-Coxe M Young T Finn L Mignot E. Depression: relationships to sleep paralysis and other sleep disturbances in a community sample. J Sleep Res. (2007) 16:297–312. 10.1111/j.1365-2869.2007.00600.x 17716279 PMC2800990

[25] McGrath PJ Stewart JW Harrison WM Ocepek-Welikson K Rabkin JG Nunes EN. Predictive value of symptoms of atypical depression for differential drug treatment outcome. J Clin Psychopharmacol. (1992) 12:197–202. 10.1097/00004714-199206000-00009 1629387

[26] Moise N Khodneva Y Richman J Shimbo D Kronish I Safford MM. Elucidating the association between depressive symptoms, coronary heart disease, and stroke in black and white adults: the REasons for geographic and racial differences in stroke (REGARDS) study. J Am Heart Assoc. (2016) 5:e003767. 10.1161/JAHA.116.003767 27521153 PMC5015296

[27] Hamieh N Meneton P Wiernik E Limosin F Zins M Goldberg M. Depression, treatable cardiovascular risk factors and incident cardiac events in the Gazel cohort. Int J Cardiol. (2019) 284:90–5. 10.1016/j.ijcard.2018.10.013 30305238

[28] Trivedi R Gerrity M Rumsfeld JS Spertus JA Sun H McDonell M. Angina symptom burden associated with depression status among veterans with ischemic heart disease. Annals Behav Med. (2014) 49:58–65. 10.1007/s12160-014-9629-4 25008432

[29] Hayek SS Ko YA Awad M Soto ADM Ahmed H. Depression and chest pain in patients with coronary artery disease. Int J Cardiol. (2017) 230:420–6. 10.1016/j.ijcard.2016.12.091 28041701 PMC5881400

[30] Li Z Yong H Han Y Wu S Zhu D Liu M. Prevalence and associated physical symptoms of depressive and anxiety symptoms in neurology outpatient clinic. J Neurol Neurosurg Psychiatr. (2019) 90:1286–7. 10.1136/jnnp-2018-320130 30760642

