# Supplementary material for: Balance Problems, Paralysis, and Angina as Clinical Markers for Severity in Major Depression
Source: Front Psychiatry. 2020 Dec 23;11:567394. doi: 10.3389/fpsyt.2020.567394 (PMC7785784; doi:10.3389/fpsyt.2020.567394)
Supplement: Supplementary file 1 [file Data_Sheet_1.doc]

**Supplementary Material**

**Supplementary Table 1.**

| **Medications** | **Low Severity** | **High Severity** |
| --- | --- | --- |
| Mirtazapine | 3% | 7% |
| Bupropion | 11% | 18% |
| Trazodone | 8% | 8% |
| Fluoxetine | 3% | 2% |
| Venlafaxine | 24% | 17% |
| Paliperidone palmitate | 3% | 0% |
| Citalopram | 18% | 5% |
| Levomilnacipran | 0% | 5% |
| Escitalopram | 6% | 2% |
| Duloxetine | 3% | 3% |
| Sertraline | 2% | 12% |
| Desvenlafaxine | 5% | 7% |
| Desipramine | 2% | 0% |
| Vortioxetine | 5% | 8% |
| Nortriptyline | 0% | 0% |
| Paroxetine | 0% | 0% |
| Amitriptyline | 2% | 7% |
| Moclobemide | 0% | 5% |
| Clomipramine | 0% | 2% |
| Tranylcypromine | 0% | 0% |
| Aripiprazole | 11% | 13% |
| Brexpiprazole | 0% | 5% |
| Lurasidone | 5% | 3% |
| Olanzapine | 2% | 2% |
| Loxapine | 0% | 2% |
| Quetiapine | 24% | 25% |
| Lorazepam | 5% | 3% |
| Clonazepam | 14% | 12% |
| Lithium | 12% | 5% |
| Methylphenidate | 9% | 13% |
| Ziprasidone | 2% | 2% |
| Lisdexamfetamine | 3% | 2% |
| Agomelatine | 0% | 2% |
| Risperidone | 2% | 2% |
| Lamotrigine | 5% | 7% |
| Dextroamphetamine and Amphetamine | 2% | 2% |
| Buspirone | 0% | 2% |
| Diazepam | 0% | 3% |
| Dextroamphetamine | 2% | 0% |
| Methotrimeprazine maleate | 2% | 0% |
| Alprazolam | 0% | 2% |
| Flupentixol decanoate | 2% | 0% |
| Oxazepam | 2% | 0% |

**Supplementary Table 1.** The percentage of patients taking each medication in the high and low severity categories was calculated. It was noted that the medications bupropion and sertraline had large differences between high and low severity categories (18% vs. 11% and 12% vs 2%, respectively). No medication side-effects were found to be related to balance problems, muscular paralysis, or angina.

**Supplementary Figure 1. A box-plot of the number of psychiatric medications taken by each patient.**


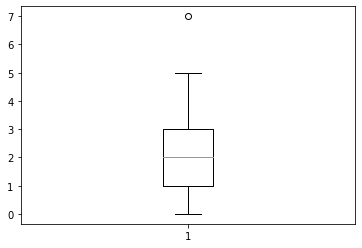


**Supplementary Figure 1.** A box-plot was generated for the number of psychiatric medications to remove outliers. One patient out of 184 had seven medications and was determined to be an outlier and removed from the subsequent linear regression analysis.

**Supplementary Figure 2. Linear regression with the number of psychiatric medications as the independent variable and the depression severity score as the dependent variable.**


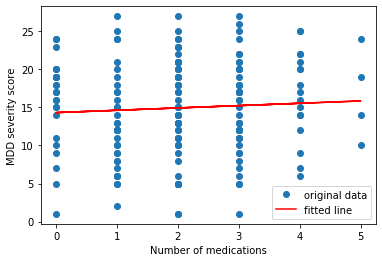


**Supplementary Figure 2.** A linear regression analysis was performed to assess the relationship between the number of psychiatric medications a patient was taking (x-axis) and their depression severity score (y-axis). The best-fit regression line has a slope of 0.3, an intercept of 14.3, an R-squared value of 0.004, and a p-value of 0.4, indicating that no significant correlation was found between the number of medications and depression severity score.
